# A population-level strain genotyping method to study pathogen strain dynamics in human infections

**DOI:** 10.1172/jci.insight.152472

**Published:** 2021-12-22

**Authors:** Sarah J. Morgan, Samantha L. Durfey, Sumedha Ravishankar, Peter Jorth, Wendy Ni, Duncan T. Skerrett, Moira L. Aitken, Edward F. McKone, Stephen J. Salipante, Matthew C. Radey, Pradeep K. Singh

**Affiliations:** 1 Department of Microbiology, University of Washington School of Medicine, Seattle, Washington, USA.; 2 Department of Pathology and Laboratory Medicine, Cedars-Sinai Medical Center, Los Angeles, California, USA.; 3 Department of Medicine, University of Washington School of Medicine, Seattle, Washington, USA.; 4 St. Vincent’s University Hospital, Dublin, Ireland.; 5 Department of Laboratory Medicine and Pathology, University of Washington School of Medicine, Seattle, Washington, USA.

**Keywords:** Infectious disease, Microbiology, Bacterial infections, Molecular genetics

## Abstract

A hallmark of chronic bacterial infections is the long-term persistence of 1 or more pathogen species at the compromised site. Repeated detection of the same bacterial species can suggest that a single strain or lineage is continually present. However, infection with multiple strains of a given species, strain acquisition and loss, and changes in strain relative abundance can occur. Detecting strain-level changes and their effects on disease is challenging because most methods require labor-intensive isolate-by-isolate analyses, and thus, only a few cells from large infecting populations can be examined. Here, we present a population-level method for enumerating and measuring the relative abundance of strains called population multi-locus sequence typing (PopMLST). The method exploits PCR amplification of strain-identifying polymorphic loci, next-generation sequencing to measure allelic variants, and informatic methods to determine whether variants arise from sequencing errors or low-abundance strains. These features enable PopMLST to simultaneously interrogate hundreds of bacterial cells that are cultured en masse from patient samples or are present in DNA directly extracted from clinical specimens without ex vivo culture. This method could be used to detect epidemic or super-infecting strains, facilitate understanding of strain dynamics during chronic infections, and enable studies that link strain changes to clinical outcomes.

## Introduction

Serial culturing of chronic infection sites often repeatedly yields the same pathogen species. For instance, chronic wounds can consistently grow *Staphylococcus* and *Pseudomonas* species (1), patients with urinary tract anomalies can be persistently infected by *Escherichia coli* (2), and chronically infected sinuses can recurrently yield the same anaerobes (1–3). The chronic infections that people with cystic fibrosis (CF) have are a prime example, as the same pathogen species are frequently cultured from patients’ lung secretions for long periods. Some species, like *Pseudomonas aeruginosa* (*P. aeruginosa*) and *Staphylococcus aureus* (*S. aureus*) can be highly abundant in the lungs of individual patients for decades or even for life (4–9).

Repeated detection of the same bacterial species over time can imply that a single strain or lineage is continually present. However, even though most strain-level genotyping studies examine very few isolates from each infection, studies on chronic wound, urinary tract, ear, gastrointestinal, and lung infections suggest more complexity. For example, strain-level genotyping methods have shown that close to a third of people with CF and *S. aureus* lung infections simultaneously harbor more than 1 *S. aureus* strain (4, 5, 10, 11). Likewise, some studies have shown that up to 40% of people with CF are simultaneously infected by 2 or more *P. aeruginosa* strains (12–14), although other work has suggested a lower frequency of multi-strain infections (7, 15–19). In addition, strain relative abundance can change over time, and strains can be gained or lost in individual patients (13, 18, 20). Notorious examples are *P. aeruginosa* epidemic strains that can infect and eventually become dominant in already colonized patients and worsen disease (21–23).

Identifying infecting strains is important for several reasons. First, strains of the same species can differ markedly in traits like the capacity for injury, transmissibility, and resistance to antibiotics (19, 22–27). Thus, the presence of multiple strains or changes in strain relative abundance could have clinical consequences. Second, strain abundance changes could provide information about the status of host defenses, treatment efficacy, or pathogen functioning. For example, strains may recede when host defenses or treatments to which they are susceptible intensify or when deleterious mutations arise. Likewise, new strain acquisition could indicate that host conditions have become more permissive, and analysis of succeeding strains could increase understanding of bacterial functions important in vivo. Third, sensitive methods for strain detection could reveal outbreaks and lapses in infection control procedures. Finally, early detection of new strains could spur eradication attempts, which may be more successful soon after strains are acquired (28–32).

Established methods for strain-level identification, such as pulse-field gel electrophoresis (PFGE), multi-locus sequence typing (MLST), and whole-genome sequencing (WGS), must generally be performed on 1 cultured isolate at a time (16, 33–36). Because pathogen populations can be extremely large and colonies from different strains may look identical (37–41), analyzing a few colonies per sample could miss multi-strain infections and strain acquisition and loss events. Newer methods using amplification of species-specific variable regions (42, 43) are not easily adaptable to multiple pathogens, and shotgun sequencing of clinical samples (41, 44) can be limited if nontarget DNA (e.g., host or other bacterial DNA) is abundant. To address these limitations, we developed PopMLST (population MLST), a method to enumerate and measure the relative abundance of strains present in pools of hundreds of cultured isolates, or in DNA directly extracted from clinical samples.

## Results

### Overview.

In conventional MLST, bacterial colonies are isolated in pure culture, Sanger sequencing is used to identify allelic variation in MLST loci within conserved housekeeping genes (7 loci in the case of *S. aureus* and *P. aeruginosa*), and loci allele types are determined by comparison to a database (45–47). Because a single clone is analyzed, the loci are known to be linked, in that they originate from the same bacterial isolate. Thus, loci allele identities can be combined to define the MLST type of a pure culture isolate.

**Figure 1:**
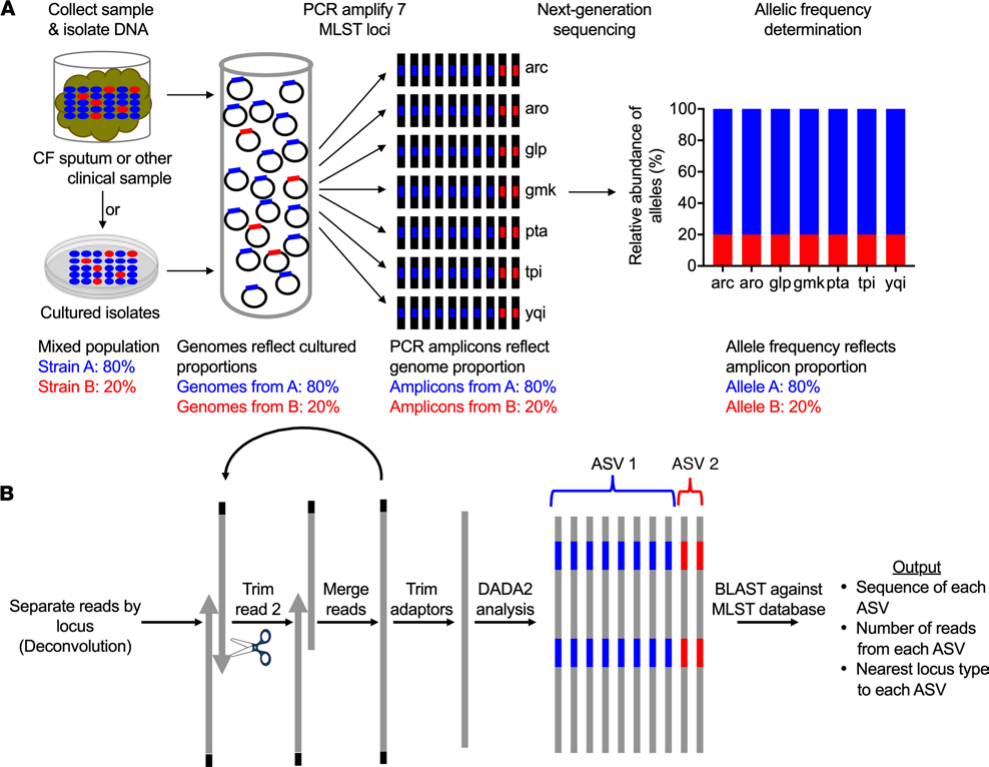
PopMLST methods. (**A**) PopMLST can be performed on clinical specimens (without culturing) or cultured isolates. MLST loci are PCR amplified in separate reactions, amplicons Illumina sequenced, and the relative abundance of reads representing MLST loci measured. (**B**) Bioinformatic analysis deconvolutes reads using permissive alignment to assign them to MLST loci, iteratively tests reverse read trimming lengths to optimize merging of paired-end reads, removes adaptors, identifies amplicon sequence variants (ASVs) using DADA2, and uses BLAST to identify the closest MLST locus type.

In contrast, the goal of PopMLST is to enumerate the pathogen strains and measure strain relative abundance in samples that could contain multiple strains, even when the infection site contains a vast excess of nontarget (e.g., human) DNA. To achieve this, PopMLST uses PCR to amplify MLST loci from complex samples and next-generation sequencing to measure allele relative abundance. The PCR primers act as probes to find conserved sequences flanking MLST loci (even when the targeted species is rare) and as vectors to amplify the strain-discriminating MLST loci. Amplicons are Illumina sequenced, and bioinformatic tools are used to distinguish rare variants from errors, group “like” sequences, and measure their relative abundance (Figure 1). A drawback to this approach is that PCR amplification and Illumina sequencing from complex mixtures are more error prone than Sanger sequencing of individual clones. Errors could be confused for low-abundance variant strains, particularly since different MLST loci can differ only at a few positions (45–47).

We addressed this problem in several ways. First, we used high-fidelity polymerases and as few PCR cycles as possible to reduce errors and PCR chimeras. Second, we adapted the DADA2 analysis pipeline for use on MLST loci amplicons. DADA2 (48) is designed for 16S rRNA amplicon sequencing and uses statistical methods to distinguish sequencing errors from low-abundance variants (Figure 1B) (43, 48). Third, we developed bioinformatic methods to adaptively trim the lower quality ends of the reverse read generated by Illumina sequencing to facilitate accurate read merging (see Methods and Figure 1B). Fourth, we amplified each MLST locus in triplicate and pooled the data to reduce random, preferential amplification of templates (i.e., “jackpot” amplifications) (49). Fifth, we omitted a GC repeat–rich *P. aeruginosa* MLST loci (*aro*) that was challenging to sequence with Illumina chemistries (Supplemental Figure 1, A–C; supplemental material available online with this article; https://doi.org/10.1172/jci.insight.152472DS1) (50, 51). Control analyses (Supplemental Figure 1, D and E) showed that omission of *aro* had only a minor effect on strain discrimination. Together, these approaches mitigate, but do not fully eliminate, the effects of PCR and sequencing errors.

### Data interpretation.

Although the PCR and Illumina sequencing used in PopMLST enable analysis of mixtures containing multiple strains and excess nontarget DNA, information from MLST loci is unlinked because many isolates are analyzed en masse and allele sequences for each locus are derived from separate PCRs. This issue does not generally limit PopMLST’s ability to enumerate and measure strain relative abundance, which can be determined by examining the loci with the highest number of alleles represented.

This approach is effective because even though strains sometimes share MLST alleles (and PopMLST will report the sum of the shared loci’s relative abundance in these cases), the large number of alleles for each locus (e.g., *S. aureus* MLST loci have 484–892 distinct alleles, and *P. aeruginosa* MLST loci have 137–278 distinct alleles) makes it unlikely that strains would have identical alleles at enough MLST loci to prevent strain enumeration. However, in mixed populations containing many strains, the likelihood that multiple strains share alleles increases, which could cause PopMLST to underestimate the number of strains present in the population.

The MLST types of strains within mixtures can also often be determined from PopMLST data. When a limited number of strains coexist, inference can determine which MLST alleles originate from the same strain, as linked alleles will be detected at a similar relative abundance. For example, if PopMLST finds that each loci contains 3 alleles at a relative abundance of ~70%:~25%:~5%, it is likely that the alleles identified at 70% relative abundance belong to one strain, alleles at 25% come from a second strain, and alleles at 5% come from a third strain. When many strains are present, if strains coexist at similar relative abundances, or if strains happen to share several alleles, inference can fail. If knowledge of the specific MLST types is important, conventional MLST can be performed on a few cultured colonies to determine which alleles are linked to one another to guide analysis of population-level data generated by PopMLST.

Once it is determined which MLST loci types likely belong to the same isolates (by inference, or by conventional MLST on single isolates), the relative abundance of each MLST type in the sample is calculated by averaging the relative abundance of all loci that differentiate the 2 strains. Averaging MLST loci abundances dampens the effect of error that could occur in individual loci measurements.

### PopMLST identifies single strains after in vivo diversification.

As an initial test of the method, we performed PopMLST on pure cultures containing single strains of *S. aureus* and *P. aeruginosa* and found that more than 99% of reads correctly reported a single MLST type in each of 21 independent experiments (Table 1).

In CF and other chronic infections, strains genetically diversify during infection (10, 19, 25–27, 52, 53), and within-strain genetic diversity could be mistaken for strain differences. Thus, we tested PopMLST on pools of 90–96 clonally related *P. aeruginosa* isolates collected from different lung regions, from 3 people with CF undergoing lung transplantation. WGS showed that isolates from each patient were clonally related to one another but had genetically diversified via in vivo evolution (19). Importantly, 2 of the 3 collections exhibited hypermutator phenotypes due to mutations in either *mutL* or *mutS* mismatch repair genes, and WGS showed that the mutator populations contained far higher levels of genetic variation than the nonmutator population (19). Core genomes of 96 isolates from the patient who was not a hypermutator contained a total 328 SNP differences, and the 96 isolates from patients with hypermutator lineages contained 3169 and 1653 SNP differences (19).

**Figure 2:**
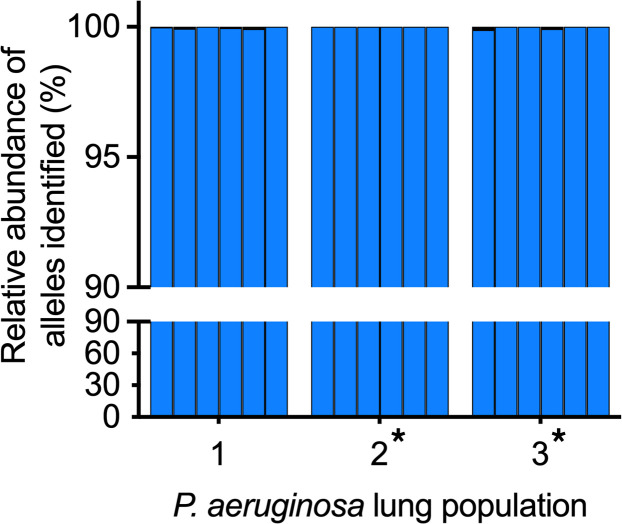
PopMLST correctly identifies genetically diversified, clonally related *P. aeruginosa* as a single MLST type. PopMLST was performed on pools of 90–96 clonally related *P*. *aeruginosa* isolates collected from different lung regions, from 3 CF patients undergoing lung transplantation. Plot shows the relative abundance of each MLST allele (from pool) that matches the known MLST sequence (determined by WGS; see Supplemental Table 1). The 6 bars for each sample show the relative abundance of *acs*, *gua*, *mut*, *nuo*, *pps*, and *trp* loci (in order). Black bars indicate any additional MLST loci types detected, which in all cases were less than 0.2%. Asterisk indicates hypermutable populations due to *mutS* (population 2) or *mutL* (population 3) mutations.

Despite this extensive evolved diversity, PopMLST correctly identified each of the populations as containing a single MLST type (<0.01% of reads erroneously reported a second MLST allele) (Figure 2 and Supplemental Table 1). These data suggest that the measures used to mitigate PCR amplification and sequencing errors are effective for pure culture isolates and diversified clonally related populations.

### PopMLST accurately measures pathogen strains in experimental mixtures.

**Figure 3:**
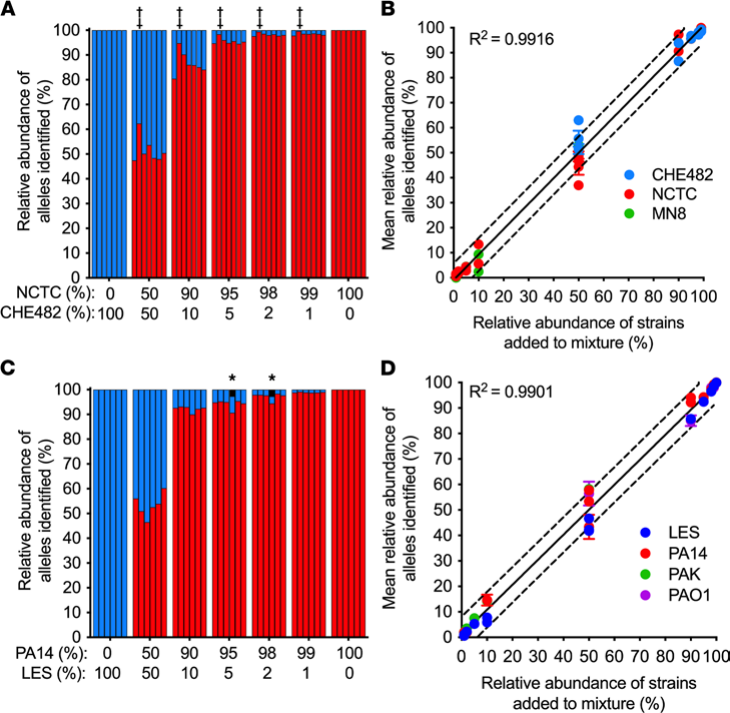
PopMLST measures strain relative abundance. (**A** and **C**) DNA from 2 *S*. *aureus* (Sa) (**A**) and *P*. *aeruginosa* (Pa) (**C**) strains with different MLST types were mixed at indicated ratios. Red bars indicate reads that PopMLST called as alleles from Sa NCTC8325 (**A**) and Pa PA14 (**C**); blue bars indicate reads called as alleles from Sa CHE482 (**A**) and Pa LES (**C**). (**B** and **D**) Mean loci relative abundance and SEM corresponding to the indicated MLST type from 21 independent 2-strain Sa mixtures (**B**), including NCTC8325 (red) with CHE482 (blue) or MN8 (green), and 20 independent 2-strain Pa mixtures (**D**), including PA14 (red) and LES (blue) or PAK (green), or PAO1 (purple) and LES (blue). Data for individual allele measurement can be found in Supplemental Figures 2 and 3. Some error bars (SEM) were smaller than symbols; solid line indicates expected result, dashed lines indicate ±10%. Bars in **A** show relative abundance of *arc*, *aro*, *glp*, *gmk*, *pta*, *tpi*, and *yqi* (in order). Bars in **C** show relative abundance of *acs*, *gua*, *mut*, *nuo*, *pps*, and *trp* (in order). MLST alleles identified but not present in the mixtures (likely sequencing error) are indicated in black, and those detected at more than 1% are indicated with *. ‡ indicates PCR bias as evidenced by 1 allele being consistently under- or overrepresented.

A key assumption of the PopMLST approach is that the relative abundance of MLST loci present in samples is maintained through DNA extraction, amplification, sequencing, and enumeration steps (Figure 1). We therefore began testing PopMLST’s ability to detect multiple strains using defined mixtures of purified DNA from different strains. PopMLST identified the expected ratios (within 2-fold) of mixtures containing 2 *S. aureus* or *P. aeruginosa* strains over a wide relative abundance range (Figure 3). Replicate experiments using different sequencing runs and different MLST types produced similar results (Figure 3 and Supplemental Figures 2 and 3). Linear regression of data from the experimental mixtures indicated close agreement between expected results and average MLST allele loci measurements (*R*^2^ = 0.9916 for *S. aureus* and *R*^2^ = 0.9901 for *P. aeruginosa*), with slopes approximating 1 (*S. aureus*: 1.017 [95% CI: 0.9872–1.047]; *P. aeruginosa*: 0.9806 [95% CI: 0.9454–1.016]).

**Figure 4:**
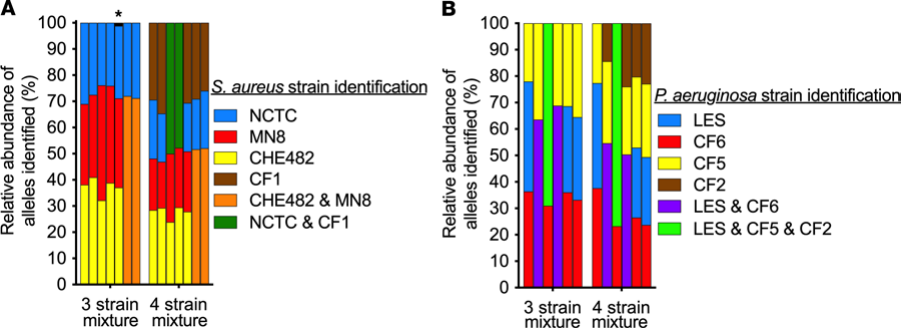
PopMLST can identify at least 4 unique MLST types in a mixture. Equimolar mixtures of DNA from 3 or 4 strains were analyzed by PopMLST. Unique MLST loci alleles of the 4 strains present in the mixtures are shown as blue, red, yellow, and brown. Alleles shared between strains added to the mixture cannot be assigned to a particular strain and are therefore colored as follows: alleles common to strains indicated with yellow and red are indicated with orange; alleles common to strains indicated with blue and brown are indicated with green (**A**); alleles common to strains indicated with red and blue are indicated with purple; and alleles common to strains indicated with blue and yellow and brown are indicated with green (**B**). Bars in **A** show relative abundance of Sa MLST alleles *arc*, *aro*, *glp*, *gmk*, *pta*, *tpi*, and *yqi* (in order). Bars in **B** show relative abundance of Pa MLST alleles *acs*, *gua*, *mut*, *nuo*, *pps*, and *trp* (in order). MLST alleles identified but not present in the mixtures (likely sequencing error) are indicated in black, and those detected at more than 1% are indicated with *.

We also tested PopMLST’s ability to measure strain relative abundance in 3- and 4-strain mixtures (Figure 4). PopMLST measurements of strain relative abundance in mixtures containing equal ratios of 3 *S. aureus* strains reported an average strain relative abundance of 33.7% (SEM 7.9%), and measurements of equal-ratio 4-strain *S. aureus* mixtures reported average strain relative abundance of 24.9% (SEM 5.57%) (Figure 4A). In equal-ratio *P. aeruginosa* 3-strain mixtures, PopMLST reported average strain relative abundance of 33.2% (SEM 9.5%). In equal-ratio 4-strain *P. aeruginosa* mixtures, PopMLST reported average strain relative abundance of 24.6% (SEM 6.0%) (Figure 4B). We have not tested mixtures containing more than 4 strains.

Despite use of triplicate PCR reactions, a single locus type was occasionally detected at higher-than-expected abundance (Figure 3 and Supplemental Figures 2 and 3). These findings are likely due to PCR bias (indicated by ‡ in Figure 3 and Supplemental Figures 2 and 3) or jackpot amplifications (indicated by ^#^ in Supplemental Figure 3). PCR bias and jackpots also occur in 16S rRNA gene measurements (54), which likewise use amplicon sequencing. However, because PopMLST averages data from 6 or 7 independently amplified loci (unlike 16S sequencing, which relies on a single locus), the effect of error in any given locus is dampened. Furthermore, loci that appear to be outliers can be interpreted in the context of others to estimate strain relative abundance. Because of these advantages, PCR bias and jackpot amplifications had little effect on strain relative abundance measurements in the experimental mixtures we tested (Figure 3, B and D, and Supplemental Figures 2 and 3).

### PopMLST has a low frequency of false-positive strain calls.

Errors inherent to PCR and Illumina sequencing could cause PopMLST to artifactually report strains that are not present. We examined control experiments containing between 1 and 4 strains of known composition (*n* = 38 for *P. aeruginosa* and *n* = 41 for *S. aureus*) to examine the effect of using different abundance thresholds to make strain presence and absence calls.

As shown in Table 2, using the criterion that a single variant locus be present at ≥1% relative abundance falsely registered the presence of a new strain in 9/49 (18%) of control experiments with *P. aeruginosa* and 7/41 (17%) of control experiments with *S. aureus*. Using the criterion that 2 or more loci be present at ≥1%, or raising the relative abundance threshold for a single allele to ≥4%, produced accurate calls in all 49 *P. aeruginosa* and all 41 *S. aureus* experiments. We conclude that using a threshold of a single variant locus at greater than 4% relative abundance or 2 variant loci at greater than 1% relative abundance results in a low likelihood of erroneously interpreting sequencing error as strain presence. Moreover, because the false-positive calls we detected tended to be sequencing run specific (Supplemental Table 2), their impact on PopMLST’s accuracy could be decreased by repeated sequencing of samples.

### PopMLST can detect specific MLST types with high sensitivity.

In certain settings, clinicians and researchers need to detect specific strains with known MLST types. Examples include superinfections with virulent *P. aeruginosa* epidemic strains in people with CF already colonized by *P. aeruginosa* or infection control surveillance during outbreaks. Theoretically, known MLST types should be detectable with higher sensitivity than unknown types, as it is extremely unlikely that the chance occurrence of errors would report the presence of the specific MLST loci of interest.

To test this, we measured PopMLST’s sensitivity to detect targeted low-abundance MLST alleles in complex mixtures. As shown in Tables 3 and 4, targeted low-abundance alleles were detected in all experiments when present at 5% relative abundance or greater and in almost all experiments when present at 2%, 1%, and 0.1% relative abundance. These findings suggest that PopMLST could be used for early detection of known strains with high transmissibility or virulence or to investigate efficacy of infection control measures.

### PopMLST works in the presence of excess human or nontarget bacterial DNA.

Clinical samples can contain vast amounts of human and nontarget bacterial DNA. For example, despite high pathogen density (*P. aeruginosa* can reach 10^8^–10^9^ CFU/mL in CF sputum), 95%–99% of CF sputum DNA is human (55), and DNA from other pathogens or oral bacteria can also be highly abundant.

**Figure 5:**
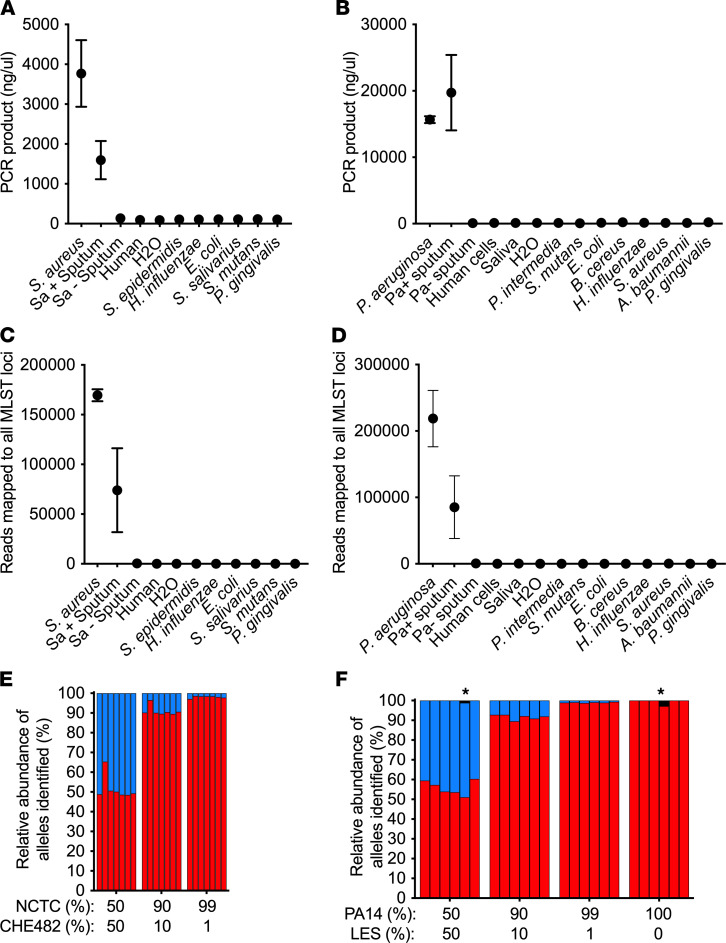
Heterologous DNA does not interfere with PopMLST. (**A** and **B**) The average concentration of PCR product from the 7 or 6 amplified loci after PCR using PopMLST primers for Sa (**A**) and Pa (**B**) on DNA from the indicated sources. “Human” indicates DNA extracted from tissue culture cells; “H2O” indicates ultrapure water; “Sa+ sputum” and “Pa+ sputum” indicate sputum from 3 patients with CF who were culture positive for Sa and Pa, respectively; and “Sa- sputum” and “Pa- sputum” indicate sputum from a CF patient who was culture negative for Sa and Pa. (**C** and **D**) The sum of sequence reads produced by PopMLST that mapped to 7 Sa (**C**) or 6 Pa (**D**) MLST loci shown for samples containing target and nontarget DNA from PCR reactions in **A** and **B**. Mixtures of Sa NCTC8325/CHE482 were used as positive control in **A** and **C**, and mixtures of PA14/PAO1 were used in **B** and **D**. For samples with negligible PCR amplification, more than 2 times volume of sample was used for Illumina sequencing than was used for other samples. The average and SEM of 3 separate samples are shown for Sa, Sa+ sputum, Pa, and Pa+ sputum in **A**–**D**. (**E** and **F**) 95% human DNA from tissue culture cells was added to the same mixtures of 2 control strains from Figure 2C. Bars in **E** show relative abundance of *arc*, *aro*, *glp*, *gmk*, *pta*, *tpi*, and *yqi* matching the MLST type of NCTC8325 (red) or CHE482 (blue). Bars in **F** show relative abundance of *acs*, *gua*, *mut*, *nuo*, *pps*, and *trp* (in order) matching the MLST type of PA14 (red) or LES (blue). Asterisk indicates the presence of an unexpected loci type (black), likely due to sequencing error.

We investigated the effects of contaminating DNA on PopMLST in 2 ways. First, we performed PCR on human and nontarget bacterial DNA (including closely related species) using *S. aureus* and *P. aeruginosa* PopMLST primers. Amplicon yields and the number of reads mapping to *S. aureus* and *P. aeruginosa* MLST loci in these experiments were similar to no-template controls (Figure 5, A–D). Second, we tested the ability of PopMLST to detect strains in the presence of 95% human DNA, and we found that the vast excess of human DNA did not compromise detection, even when strains were present at as low as 1% relative abundance (Figure 5, E and F).

### PopMLST measures strain abundance in clinical samples.

**Figure 6:**
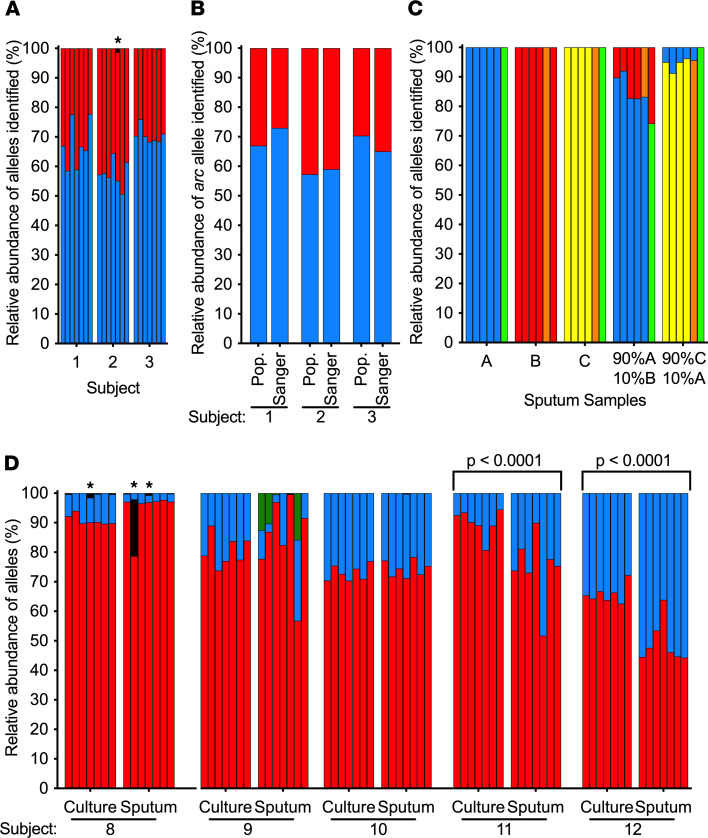
PopMLST on clinical samples. (**A** and **B**) PopMLST results from 3 of 7 patients with CF in whom PopMLST detected 2 Sa strains (see Supplemental Figure 4 for the 4 of 7 patients in whom 1 strain was detected). (**A**) PopMLST results from DNA pooled from 90–95 cultured Sa isolates. Blue and red bars indicate different MLST loci alleles for each patient (Supplemental Table 3). Bars for each sample show relative abundance of *arc*, *aro*, *glp*, *gmk*, *pta*, *tpi*, and *yqi*. A third *pta* allele (black bar and indicated with *) differed at a single nucleotide from the allele indicated in red, likely representing sequencing error or mutation. (**B**) Relative abundance of *arc* locus as measured by PopMLST (Pop) and by individually Sanger sequencing (Sanger) 20–30 isolates from each sample from **A**. (**C**) PopMLST performed directly on DNA isolated from 3 sputum samples, A–C, and from mixtures of these samples. Red, blue, and yellow bars indicate the abundance of MLST alleles corresponding to samples A–C, respectively; green bars indicate an allele shared between samples A and C; and orange bars indicate an allele shared between samples B and C (see Supplemental Table 1). Control experiments examining more than 100 Pa isolates cultured from sputum samples A–C showed each contained a single Pa MLST type. (**D**) PopMLST of DNA pooled from more than 100 Sa cultured colonies (culture) and directly from sputum (sputum). Red and blue indicate different MLST types, which were confirmed by Sanger sequencing individual isolates (Supplemental Table 1). Subject 9’s sputum contained 3 loci with an additional allele, likely indicating a third MLST type (green) that was not detected in culture. Indicated significant differences were determined by the multiple *t* test of minor allele abundance. Black bar indicated with * indicates the presence of a third allele, likely due to mutation or sequencing error.

Encouraging results with experimental strain mixtures led us to perform proof-of-principle tests of PopMLST on clinical samples. In the first test, we cultured sputum from 7 *S. aureus*-infected CF patients and performed PopMLST on DNA prepared from about 100 colonies that grew from each sample (scraped en masse from culture plates). PopMLST reported that 3 of 7 samples contained 2 *S. aureus* MLST types (Figure 6A, Supplemental Figure 4, and Supplemental Table 3). We verified the presence of 2 strains in these samples by Sanger sequencing a distinguishing MLST locus in 20–30 individual colonies from each sample and found MLST types at a similar relative abundance as determined by PopMLST (*R*^2^ = 0.9247; slope = 0.9296 [95% CI: 0.5612–1.298]) (Figure 6, A and B).

Second, we tested PopMLST’s ability to resolve strains in CF sputum by mixing sputum samples that contained a single strain as determined by PopMLST (>99% of reads from each loci reporting a single allele) at varying ratios. When samples were combined, MLST types were identified in proportions close to the expected ratios (Figure 6C and Supplemental Table 1).

Finally, we analyzed sputum samples from 5 patients who were all known to harbor 2 *S. aureus* strains each. We performed PopMLST on DNA prepared directly from sputum and from several hundred *S. aureus* isolates from each sample scraped en masse from culture plates. As shown in Figure 6D, PopMLST detected the same MLST loci types in sputum DNA and culture scrapes from all 5 patients. Conventional MLST of representative isolates confirmed the dominant and secondary sequence types PopMLST detected (Figure 6D and Supplemental Table 3). In patients 11 and 12, strain relative abundance differed in sputum as compared with the culture scrapes (*P* < 0.001 by multiple *t* test), and in patient 9, a third MLST loci type was detected in sputum that was not present in ex vivo culture. These findings could be due to differential growth capacity of strains in ex vivo culture conditions.

## Discussion

Infecting bacteria can exhibit heritable diversity at the species, strain, and intra-strain level. While recent findings and new methods have accelerated work on species diversity (56–64) and diversification within individual strains (10, 19, 25–27, 52, 53), studies of strain-level diversity have lagged. A major factor limiting progress is that established methods to detect strains must generally be performed on one cultured isolate at a time (16, 33–36). Thus, nondominant strains are difficult to detect.

PopMLST addresses this problem because it can estimate the relative abundance of strains in pools of tens to hundreds of isolates that have been cultured en masse from infected sites and can be used on DNA extracted directly from clinical specimens without prior culture. Other advantages include technical approaches to minimize PCR and sequencing error, its robustness when human or other bacterial DNA is in vast excess (Figure 5), its ability to detect targeted strains at low relative abundance (Tables 3 and 4), and its ability to detect strains with ex vivo growth defects when the method is used on DNA prepared directly from clinical specimens. Furthermore, the method is accurate even when intra-strain genetic diversity has evolved (Figure 2 and Supplemental Table 1), likely because MLST loci are within conserved “housekeeping” genes that may be less variable than elements involved in Spa typing (6), Random Amplified Polymorphic DNA, or PFGE (16, 33). Finally, MLST databases are in widespread use and exist for over 100 bacterial species (47), so PopMLST can easily be adapted for use with many organisms with results being comparable between laboratories.

PopMLST also has limitations. First, several circumstances can limit PopMLST’s ability to enumerate and identify strains. These include the presence of unrelated strains having the same MLST sequence type, *P. aeruginosa* strain mixtures in which *aro* is the only distinguishing locus (see Supplemental Figure 1), strain mixtures in which most MLST alleles are shared between strains, or cases where evolved diversity substantially alters MLST loci. However, these circumstances will be relatively rare because many MLST types have been identified for most pathogens. For example, about 3500 *P. aeruginosa* and about 5500 *S. aureus* MLST types have been described to date (47), so strain-distinguishing power is generally robust. Moreover, MLST loci are generally conserved, and our control experiments showed PopMLST accurately identified mutator strains that had genetically diversified in vivo (Figure 2).

Second, while PCR and Illumina sequencing enable the method to be used on complex mixtures containing multiple strains and abundant nontarget DNA, these techniques are subject to errors and biases. We reduce, but cannot entirely eliminate, the effect of these problems using replicate PCR, adaptive trimming, and statistical methods.

Third, PopMLST does not identify which MLST loci are linked in individual isolates. Although this limitation does not compromise strain enumeration and relative abundance measurements under most circumstances, it can complicate identification of the strain types present in complex mixtures. Finally, we have not yet performed head-to-head tests comparing PopMLST’s resolution with other available methods for strain enumeration.

Despite these limitations, we think that PopMLST could provide valuable strain-level information in several settings. One key area is in infection control, as PopMLST’s ability to detect strains of interest at low relative abundance is advantageous, particularly if the strain being tracked belongs to a commonly encountered species. PopMLST performed serially on clinical samples could also detect episodes of strain gain or loss in individual patients. Strain changes could herald important variation in the host environment (e.g., changes in host defenses or treatments that select for new strains) or mutational changes in existing strains that compromise strain persistence in vivo. Additionally, PopMLST can detect strains with ex vivo growth defects.

PopMLST could also be used for early detection of superinfections with highly virulent or antibiotic-resistant strains. A notorious example is the Liverpool epidemic strain of *P. aeruginosa*, which can displace existing *P. aeruginosa* strains that infect the lungs of people with CF and cause increased morbidity and treatment resistance (65). Since the discovery of the Liverpool strain, other epidemic strains have been identified at disparate locations worldwide (24). Early detection of such strains is difficult in already colonized patients because clinical microbiology analyses only report the species present, and detecting epidemic strains when they are at low relative abundance would require tests on tens or hundreds of cultured isolates using conventional colony-by-colony assays (like MLST).

Finally, PopMLST could be used to investigate strain changes not associated with epidemic strains. Patients with chronic bacterial infections frequently experience highly variable disease manifestations, antibiotic responses, and rates of progression. The acquisition of different strains of a given species or changes in strain relative abundance could account for some of this variation (66). PopMLST will enable new hypotheses that explore the effects of strain-level diversity on human infection to be tested.

## Methods

### Patient samples.

*S. aureus* was isolated after sputolysin-diluted sputum was cultured on Mannitol Salt Agar (Difco). Populations were scraped from plates containing more than 100 colonies by flooding the plate with 2 mL of LB and using an L-spreader to resuspend the bacteria. *P. aeruginosa* was isolated after sputolysin-diluted sputum was cultured on MacConkey (Difco). All cultures were stored at –80°C in 15% glycerol prior to analysis. Sputum samples analyzed directly were diluted with sputolysin as above and stored at –80°C until DNA isolation could be performed.

### DNA isolation.

*P. aeruginosa* DNA was isolated from 100 μL of resuspended culture using the DNeasy Blood and Tissue kit (QIAGEN) using the protocol for Gram-negative bacteria. Due to the difficulty of lysing Gram-positive bacteria, all *S. aureus* and sputum samples were extracted using methods with increased lysis efficacy. DNA extraction of *S. aureus* and sputum samples was performed using the DNeasy PowerSoil Pro Kit (QIAGEN) with the following modifications: samples were incubated with 2.9 mg lysozyme and 0.14 mg lysostaphin prior to lysis with 0.1 mm beads using a bead beater (Mini-Beadbeater-16; Biospec), a method validated previously by our laboratory for efficient *P. aeruginosa* and *S. aureus* lysis from sputum (41), or using the DNeasy PowerSoil Pro Kit for the Qiacube automated extraction system, which results in comparable lysis of *S. aureus* and *P. aeruginosa* from sputum in our laboratory. The lysis method used depended on the availability of the extraction method at the time of sample collection. All samples were eluted in Buffer EB (QIAGEN) regardless of extraction method.

### Control mixtures.

Control strains (Supplemental Tables 4 and 5) were streaked from freezer stocks and grown overnight prior to DNA isolation. *S. aureus* and *P. aeruginosa* strains were from the Singh lab as well as donated by Matthew Parsek (University of Washington, Seattle, Washington, USA) and Lucas Hoffman (University of Washington, Seattle, Washington, USA). Cultured HELA or HEK293 cells (donated by Joseph Mougous, University of Washington, Seattle, Washington, USA) were pelleted and DNA was isolated as above. Isolated DNA was quantified by Qubit and mixed at ratios described in the figures. MLST types of control strains were based on the MLST database and confirmed by MLST typing of single isolates if necessary (Supplemental Tables 4 and 5). PAO1-lacZ:PA14 mixtures were premixed at designated ratios and plated on LB+xGal to confirm the ratio. Growth from the plate was scraped and subjected to DNA isolation as above.

### PopMLST amplification and sequencing methods.

A total of 5 ng/μL DNA from cultured bacteria, 20 ng/μL of sputum DNA, or 20 ng/μL of bacterial DNA mixed with human DNA was amplified by PCR using published MLST primers for *S. aureus* and *P. aeruginosa* (45, 46) with Illumina adaptors on the 5′ ends to enable next-generation sequencing of MLST loci (Supplemental Table 6). PCR amplification of each of the 7 MLST loci was performed in triplicate to reduce chances of random PCR bias using reagents listed in Supplemental Table 7. Triplicate reactions were pooled after PCR, and amplified DNA was visualized by agarose gel electrophoresis and quantified by Pico green (Thermo Fisher Scientific). After cleaning with Ampure beads (Beckman Coulter), the 7 MLST loci for each sample were pooled in equimolar amounts and barcoded with Illumina Nextera XT indexes. PCR amplification, indexing, and cleanup was performed as described in the 16S Metagenomic Sequencing Library Preparation guide (Illumina). Indexed MLST loci were cleaned with Ampure beads (Beckman Coulter) prior to pooling and sequenced on the Illumina MiSeq to produce 2 × 300 bp paired-end reads.

### Bioinformatic analysis.

Methods outlined below for PopMLST are available at https://github.com/marade/PopMLST (commit ID 0de7f83). Because DADA2 is designed for 1 amplicon locus (typically 16S) at a time, reads were deconvolved based on their locus-specific primer sequence using Python tre, with approximate matching to MLST loci allowing for up to a 25% mismatch (https://github.com/laurikari/tre/; commit ID 6092368) before analysis. Due to a large number of paired-end reads failing to merge for some loci, we developed a dynamic read-trimming method using a binary search algorithm, which iteratively trims the 3′ end of the reverse read and retests merging until it maximizes the number of merged reads of the correct size. Trimmed reads for all MLST loci, except *yqi*, were merged using VSEARCH 2.13.4 fastq_mergepairs. Two base pairs of the sequence in the *yqi* locus beyond the 3′ ends of reads 1 and 2 (due to the length of this amplicon) were artificially supplied (these bases are conserved according to the MLST database; ref. 47). *yqi* reads were joined using VSEARCH 2.13.4 fastq_join. Merged reads, with their adaptors trimmed using Cutadapt 2.3, were then processed using the remaining standard DADA2 pipeline steps to generate ASVs for each locus.

To determine the identity and quantify the relative abundance of each MLST locus, the ASVs were queried against the PubMLST database (https://pubmlst.org/saureus/ and https://pubmlst.org/paeruginosa/) (47) for the appropriate species using BLAST+ BLASTN (67). The matching sequence with the highest identity and longest length (less than or equal to the maximum locus length present in the database) was used to label each ASV by locus type, with less than 100% identity matches being marked as potentially novel alleles. The resulting output table includes each MLST loci type identified, the ASV, and the number of reads assigned to each type, much like a classic 16S operational taxonomic unit table.

### Statistics.

The abundance of MLST types was reported as the average relative abundance of 7 corresponding *S. aureus* MLST loci or 6 corresponding *P. aeruginosa* MLST loci, and standard error of the mean was reported. When strain type(s) were known (Figures 2 and 3 and Table 1), the relative abundances of the alleles matching that strain type were used. When strain types were unknown, alleles with similar relative abundances were assumed to be from the same strain. Linear regression of the average relative abundance was used to determine the accuracy of measuring strain abundance (Figure 3, B and D). *R*^2^ values were reported in text. Multiple *t* tests were used to compare relative abundance of individual loci from sputum with those from bacterial culture (Figure 6D). *P* values more than 0.05 were determined to be not significant and were not reported. All statistical analyses were performed in GraphPad Prism.

### Study approval.

Sputum samples were collected in accordance with the University of Washington Institutional Review Board (approved protocol numbers 06-4469 and STUDY00011983) and the Research Ethics Committee at St. Vincent’s University Hospital, Dublin, Ireland (RS20-048). Patients provided written informed consent prior to collection of samples.

## Author contributions

SJM, SLD, and PKS conceived the study; SJM, SLD, and MCR designed the methodology; MCR developed the software; SJM, SLD, and WN validated data; PKS, SJM, SLD, SR, and DTS investigated; PKS, MLA, SJS, PJ, and EFM provided resources; SJM, SLD, and PKS wrote the manuscript; PKS supervised; and PKS acquired funding. All authors have read and agreed to the published version of the manuscript.

## Supplementary Material

Supplemental data

## Figures and Tables

**Table 1:**
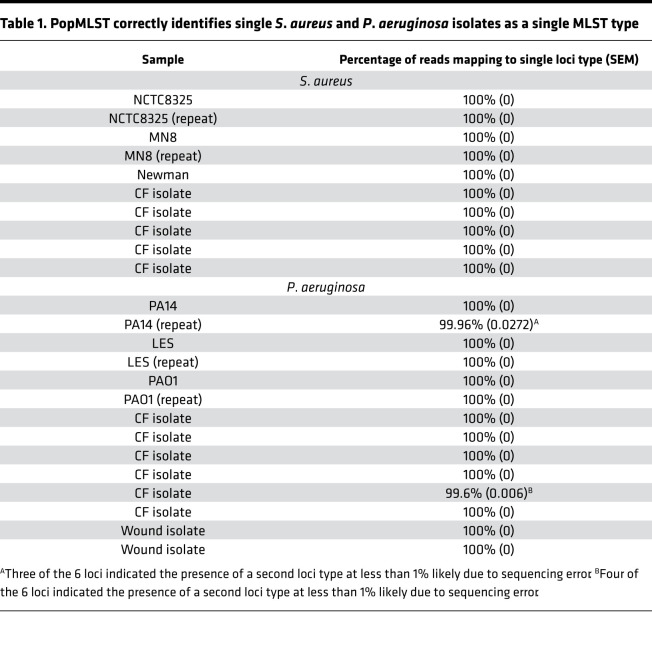
PopMLST correctly identifies single *S*. *aureus* and *P*. *aeruginosa* isolates as a single MLST type

**Table 2:**
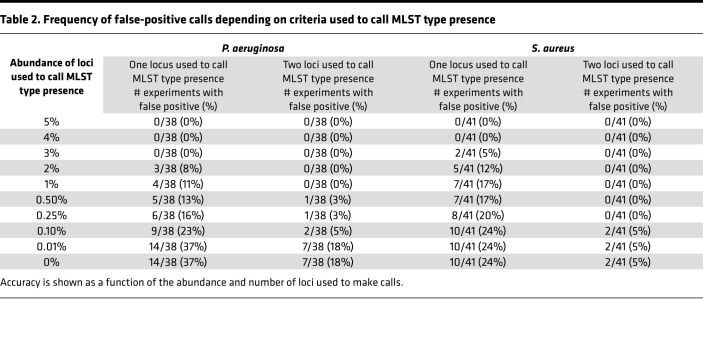
Frequency of false-positive calls depending on criteria used to call MLST type presence

**Table 3:**
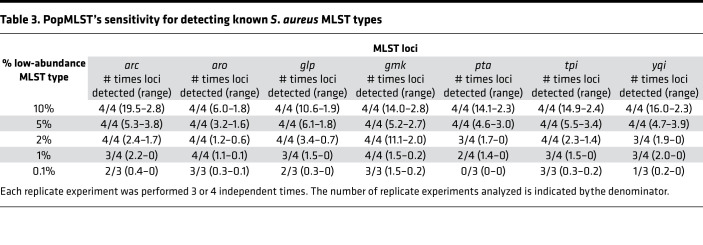
PopMLST’s sensitivity for detecting known *S*. *aureus* MLST types

**Table 4:**
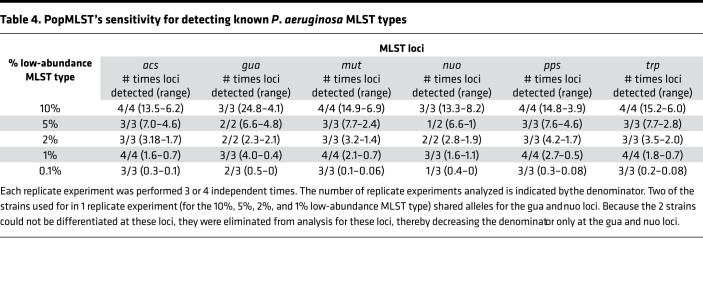
PopMLST’s sensitivity for detecting known *P*. *aeruginosa* MLST types

## Version 1 12/22/2021

Electronic publication
