# Supplementary material for: A population-level strain genotyping method to study pathogen strain dynamics in human infections
Source: JCI Insight. 2021 Dec 22;6(24):e152472. doi: 10.1172/jci.insight.152472 (PMC8783678; doi:10.1172/jci.insight.152472)
Supplement: Supplemental data [file jciinsight-6-152472-s011.pdf]

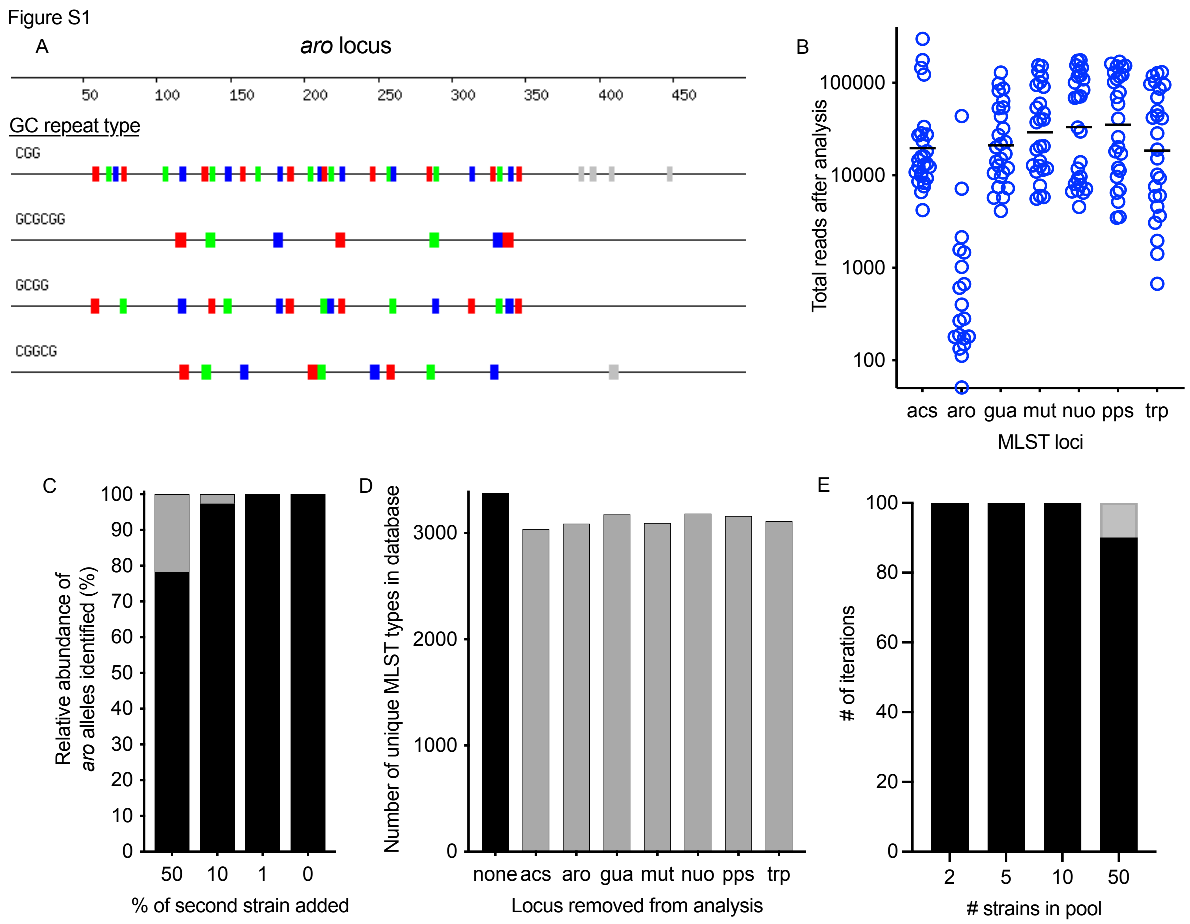

Figure S1. *P. aeruginosa aro* MLST loci are underrepresented by PopMLST. A. Schematic representation of high GC repeats in the *aro* MLST locus with each box showing the location of repeats identified by Geneious Prime 2020.1.1 (https://www.geneious.com). **B.** The number of reads recovered from *aro* are more than a log lower than other loci despite four-times higher levels of DNA used as input for Ilumina sequencing. **C.** Despite the low number of reads as shown in B, the *aro* reads detected were in ratios are similar to the expected ratios of the strains and the ratios of the other loci shown in Figure 3C. **D.** Analysis of the 3391 MLST types in the *P. aeruginosa* MLST database shows that omitting any one of the seven MSLT loci (indicated on the x-axis) still enables most MLST types to be identified. The number of unique combinations of MLST allele types when all seven alleles were utilized is indicated in black. The alleles for a given locus were removed and the analysis for unique combinations of allele types using the remaining six loci were determined (grey bars). **E.** Sets of two, five, ten, or 50 MLST types were randomly selected from the MLST database (https://pubmlst.org/organisms/pseudomonas-aeruginosa/) and the sets were used to determine if the other six loci (i.e. omitting *aro*) was sufficient to distinguish between the strains in the set. This analysis was repeated 100 times. Sets for which all MLST types could be distinguished without *aro* are shown in black. Sets for which *aro* was required for differentiation between MLST types in the set are in grey.

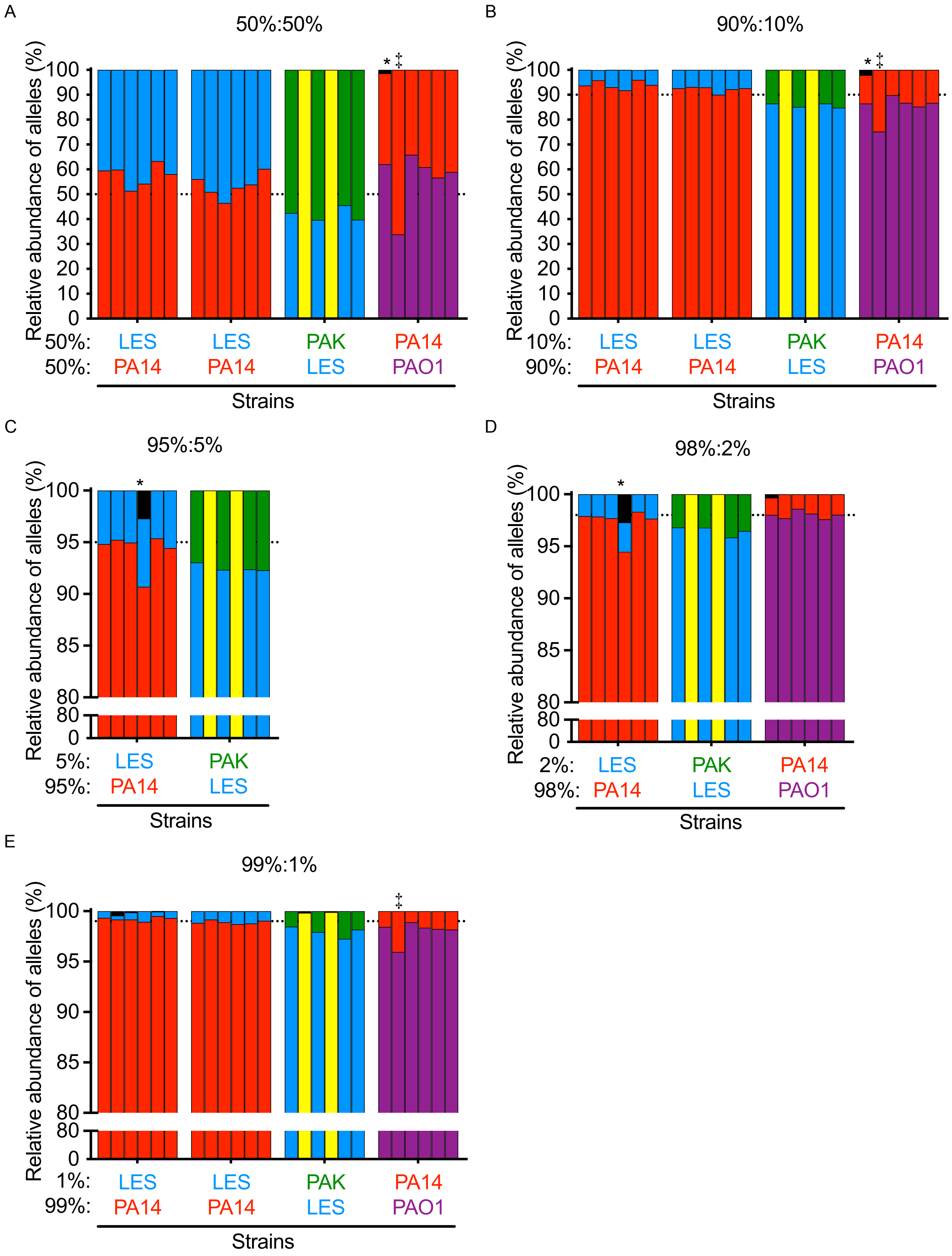

A
50%:50%
Relative abundance of alleles (%)
100
90
80
70
60
50
40
30
20
10
0
* ‡
50%: LES LES PAK PA14
50%: PA14 PA14 LES PAO1
Strains
B
90%:10%
Relative abundance of alleles (%)
* ‡
10%: LES LES PAK PA14
90%: PA14 PA14 LES PAO1
Strains
C
95%:5%
Relative abundance of alleles (%)
100
95
90
85
80
80
0
*
5%: LES PAK
95%: PA14 LES
Strains
D
98%:2%
Relative abundance of alleles (%)
*
2%: LES PAK PA14
98%: PA14 LES PAO1
Strains
E
99%:1%
Relative abundance of alleles (%)
‡
1%: LES LES PAK PA14
99%: PA14 PA14 LES PAO1
Strains

Figure S2. PopMLST on mixtures of Pa laboratory strains reproducibly identifies the relative abundance of each strain. We tested pairwise combinations of four Pa strains with different MLST types across a range of ratios. In all cases, the two strains were identified in ratios close to expected. Each strain is represented by a different color (PAO1=purple, PA14=blue, LES=red, and PAK=green). PA14 and PAK have the same sequence for two loci indicated in yellow. PopMLST was performed on mixtures of cultured colonies for the PAO1:LES experiments, all others were performed on mixtures of DNA. Each set of bars represents an independent experiment. MLST alleles identified but not present in the mixtures, likely due to sequencing error, are indicated in black and those detected at >1% are indicated with *. ‡ indicates PCR bias as evidenced by one allele being consistently under- or over-represented.

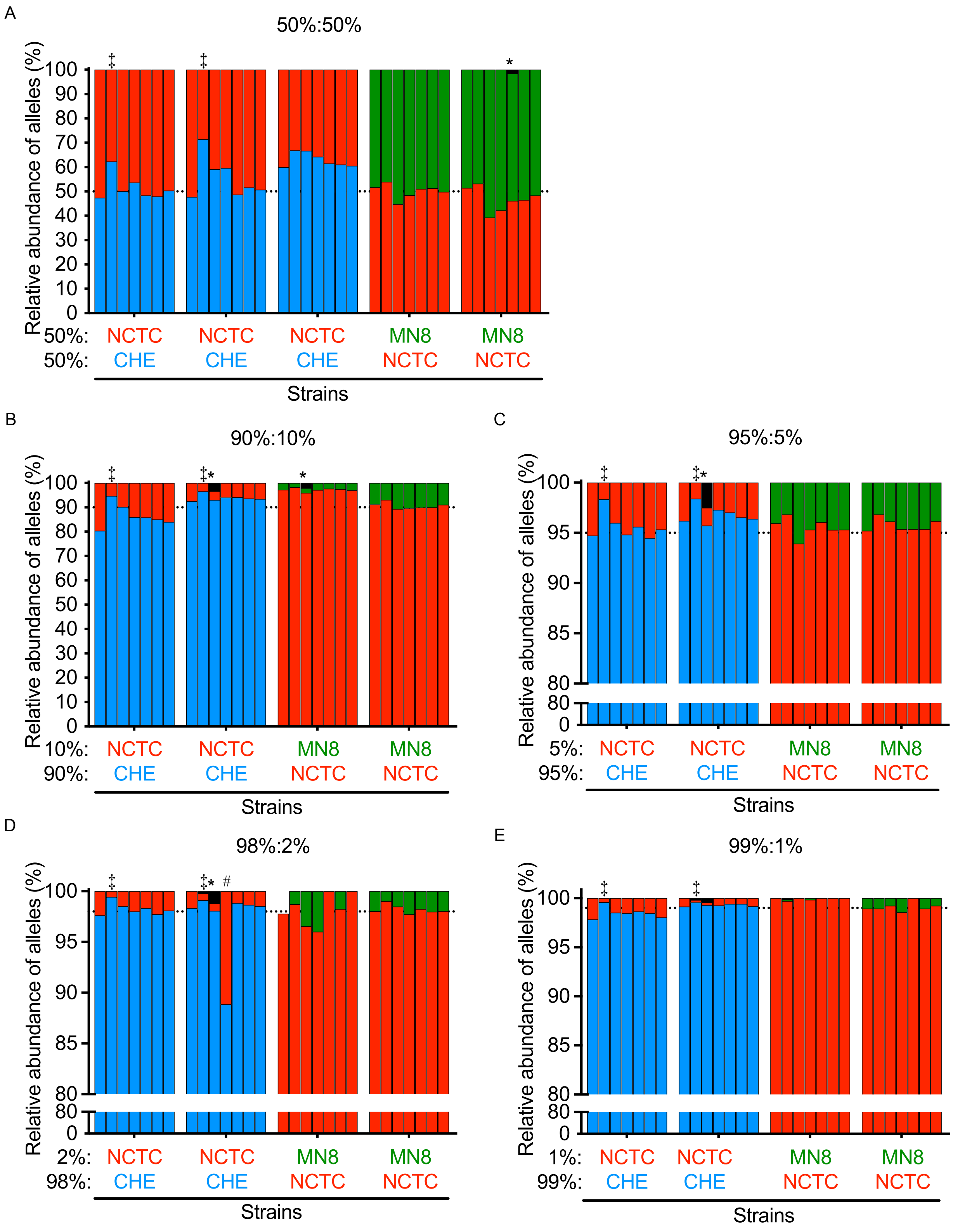
A
50%:50%
Relative abundance of alleles (%)
100
90
80
70
60
50
40
30
20
10
0
‡
‡
*
50%: NCTC NCTC NCTC MN8 MN8
50%: CHE CHE CHE NCTC NCTC
Strains
B
90%:10%
Relative abundance of alleles (%)
‡
‡*
*
10%: NCTC NCTC MN8 MN8
90%: CHE CHE NCTC NCTC
Strains
C
95%:5%
Relative abundance of alleles (%)
100
95
90
85
80
80
0
‡
‡*
5%: NCTC NCTC MN8 MN8
95%: CHE CHE NCTC NCTC
Strains
D
98%:2%
Relative abundance of alleles (%)
‡
‡*
#
2%: NCTC NCTC MN8 MN8
98%: CHE CHE NCTC NCTC
Strains
E
99%:1%
Relative abundance of alleles (%)
‡
‡
1%: NCTC NCTC MN8 MN8
99%: CHE CHE NCTC NCTC
Strains

Figure S3. PopMLST on mixtures of Sa laboratory strains reproducibly identifies the relative abundance of each strain. We tested pairwise combinations of three Sa strains with ratios close to expected. Each strain is represented by a different color (NCTC8325=red, CHE=blue, MN8=green). Each set of bars represents an independent experiment. Black indicates the presence of a third allele. MLST alleles identified but not present in the mixtures, likely due to sequencing error, are indicated in black and those detected at >1% are indicated with *. ‡ indicates PCR bias as evidenced by one allele being consistently under- or over-represented. # indicates non-systemic under/over representation of an allele, possibly due to jackpot amplifications.

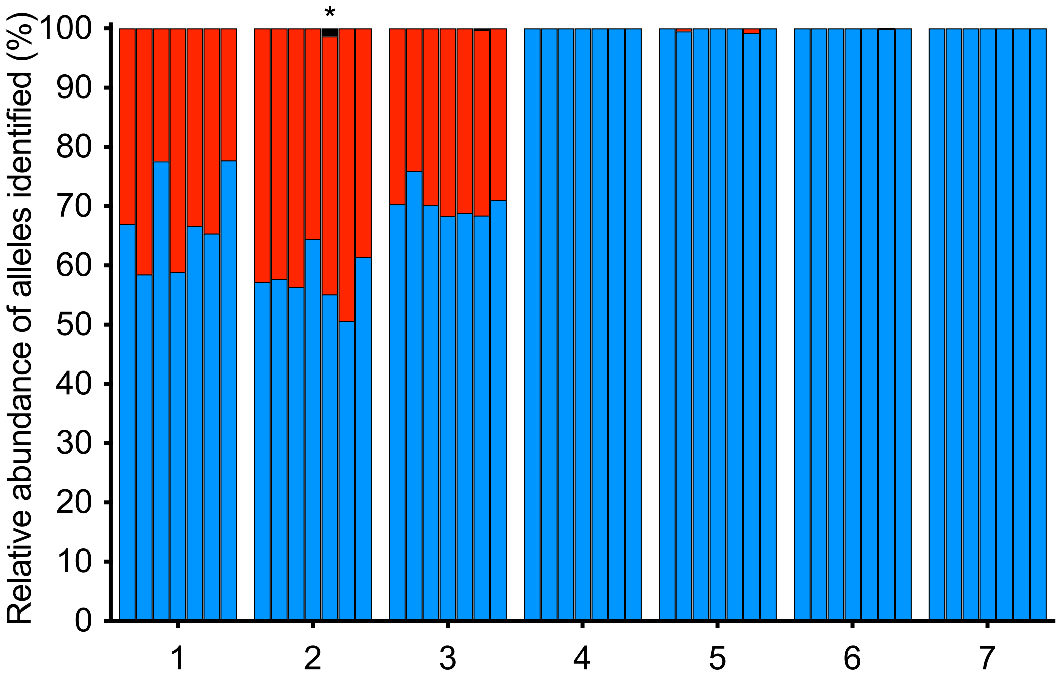

Figure S4. Detection of multiple MLST loci types from isolates from sputum. A) ~95 Sa isolates cultured on mannitol salts agar from sputum were selected from each subject. Growth from all ~95 isolates was pooled and popMLST performed. Blue indicates the predominant MLST locus type by abundance, and red indicates any secondary alleles detected. Subjects 1-3 are shown in Fgure 6. Bars for each sample show relative abundance for *arc, aro, glp, gmk, pta, tpi,* and *yqi* (in order). * indicates an additional MSLT type detected, with a single SNP, likely due to sequencing error.

Table S1. ***P. aeruginosa*** **clinical sample MLST types.**

Subject [a]	acs	gua	mut	nuo	pps	trp
1	36	28	3	4	13	7
2	6	5	3	4*	15	7
3	28	22	18*	3	15	7
A	11	1	7	9	4	7
B	17	5	3	4	15	26
C	28	22	18	3	15	7

[a] Subject number corresponds to subjects in Figures 2 and 6C.

* Number indicated is the allele type with the highest identity in the MLST database.

Table S2. False positive detection of MLST loci is eliminated if PopMLST is repeated. Sequencing errors leading to false-positive calls of a Sa or Pa MLST locus (i.e calling a locus not present in the experimental mixture) were not detected when PopMLST was repeated a second and third time.

			Proportion of samples in which false positive locus was detected (at > 1%).		
Strain mixtures tested	Expected loci type	False loci type detected (at > 1%)	In run that false positive was detected	In repeat sequencing run #1	In repeat sequencing run #2
Sa NCTC+ Sa CHE	glp_8 glp_1	glp_23	3/7	0/5	0/2
Sa MN8 + Sa NCTC	glp_1 glp_2	glp_6x	1/5	0/6	0/2
	pta_4 pta_6	pta_495	3/5	0/6	0/2
Pa PA14+ Pa LES	nuo_1 nuo_4	nuo_23	2/5	0/4	0/3

* Mixtures of the same two strains at any ratio were grouped for this analysis.

Table S3. ***S. aureus*** **clinical sample MLST types.**

subject [a]	arc	aro	glp	gmk	pta	tpi	yqi	MLST[b]
1	3	35	19	2	20	26	39	398
	1	1	1	1	1	1	1	1
2	2	2	2	2	6	3	2	30
	3	3	1	1	1	1	10	9
3	2	2	2	2	2	2	2	39
4	2	2	2	2	6	3	2	30
	5	4	1	4	4	6	3	7
5	13	13	1	1	12	11	13	15
6	10	14	8	6	10	3	2	45
7	4	1	4	1	5	5	4	25
8	3	3	1	1	4	4	3	8
	2	2	2	2	6	3	2	30
9	1	4	1	4	12	1	10	5
	2*	2	2	2	6	3	2	-
10	1	4	1	4	12	1	10	5
	10	14	8	6	10	37	2	256
11	1	4	1	4	6	1	10	-
	2	2	2	2	12	3	10	-
12	10	14	8	6	10	3	2	45
	1	4	1	4	12	1	28	105

[a] Subject number corresponds to subjects in Figures 6A, 6D, and S4.
[b] When two strains are present, MLST type determined by relative abundance of alleles and confirmed by sanger sequencing of individual isolates.
– Indicates this MLST type is not in the database.
* Number indicated is the allele type with the highest identity in the MLST database.

Table S4. ***S. aureus*** **strains and MLST types**

Strain	arc	aro	glp	gmk	pta	tpi	yqi	MLST type
NCTC8325	3	3	1	1	4	4	3	8
CHE482	10	14	8	6	10	3	2	45
MN8	2	2	2	2	6	3	2	30
CF*	13	13	1	1	12	11	13	15

* Clinical isolate is from patient with Cystic Fibrosis.

Table S5. ***P. aeruginosa*** **strains and MLST types**

Strain	acs	aro	gua	mut	nuo	pps	trp	MLST type
PA14	4	4	16	12	1	6	3	253
LES	6	5	11	3	4	23	1	146
PAO1	7	5	12	3	4	1	7	549
PAK	11	5	11	11	4	4	14	693
CF5*	28	5	30	3	3	4	14	1538
CF6*	40	5	11	5	4	38	37	167
CF2*	11	76	5	3	61	14	3	485

* Clinical isolate is from patient with Cystic Fibrosis.

Table S6. Primers used for PopMLST

MLST loci	Published MLST primer	PopMLST primer
SaarcF	TTGATTCACCAGCGCGTATTGTC	TCGTCGGCAGCGTCAGATGTGTATAAGAGACAGCCGTTGATTCACCAGCGCGTATTGTC
SaarcR	AGGTATCTGCTTCAATCAGCG	GTCTCGTGGGCTCGGAGATGTGTATAAGAGACAGGCTAGGTATCTGCTTCAATCAGCG
SaaroF	ATCGGAAATCCTATTTCACATTC	TCGTCGGCAGCGTCAGATGTGTATAAGAGACAGCCGATCGGAAATCCTATTTCACATTC
SaaroR	GGTGTTGTATTAATAACGATATC	GTCTCGTGGGCTCGGAGATGTGTATAAGAGACAGGCTGGTGTTGTATTAATAACGATATC
SaglpF	CTAGGAACTGCAATCTTAATCC	TCGTCGGCAGCGTCAGATGTGTATAAGAGACAGCCGCTAGGAACTGCAATCTTAATCC
SaglpR	TGGTAAAATCGCATGTCCAATTC	GTCTCGTGGGCTCGGAGATGTGTATAAGAGACAGGCTTGGTAAAATCGCATGTCCAATTC
SagmkF	ATCGTTTTATCGGGACCATC	TCGTCGGCAGCGTCAGATGTGTATAAGAGACAGCCGATCGTTTTATCGGGACCATC
SagmkR	TCATTAACTACAACGTAATCGTA	GTCTCGTGGGCTCGGAGATGTGTATAAGAGACAGGCTTCATTAACTACAACGTAATCGTA
SaptaF	GTTAAAATCGTATTACCTGAAGG	TCGTCGGCAGCGTCAGATGTGTATAAGAGACAGCCGGTTAAAATCGTATTACCTGAAGG
SaptaR	GACCCTTTTGTTGAAAAGCTTAA	GTCTCGTGGGCTCGGAGATGTGTATAAGAGACAGGCTGACCCTTTTGTTGAAAAGCTTAA
SatpiF	TCGTTCATTCTGAACGTCGTGAA	TCGTCGGCAGCGTCAGATGTGTATAAGAGACAGCCGTCGTTCATTCTGAACGTCGTGAA
SatpiR	TTTGCACCTTCTAACAATTGTAC	GTCTCGTGGGCTCGGAGATGTGTATAAGAGACAGGCTTTTGCACCTTCTAACAATTGTAC
SayqiF	CAGCATACAGGACACCTATTGGC	TCGTCGGCAGCGTCAGATGTGTATAAGAGACAGCCGCAGCATACAGGACACCTATTGGC
SayqiR	CGTTGAGGAATCGATACTGGAAC	GTCTCGTGGGCTCGGAGATGTGTATAAGAGACAGGCTCGTTGAGGAATCGATACTGGAAC
PaacsF	GCCACACCTACATCGTCTAT	TCGTCGGCAGCGTCAGATGTGTATAAGAGACAGCCGGCCACACCTACATCGTCTAT
PaacsR	AGGTTGCCGAGGTTGTCCAC	GTCTCGTGGGCTCGGAGATGTGTATAAGAGACAGAGGTTGCCGAGGTTGTCCAC
PaaroF	ATGTCACCGTGCCGTTCAAG	TCGTCGGCAGCGTCAGATGTGTATAAGAGACAGCCAATGTCACCGTGCCGTTCAAG
PaaroR	GTTCCTTGGCTGACGGAAGT	GTCTCGTGGGCTCGGAGATGTGTATAAGAGACAGGGTTGAAGGCAGTCGGTTCCTTG
PaguaF	AGGTCGGTTCCTCCAAGGTC	TCGTCGGCAGCGTCAGATGTGTATAAGAGACAGCCAGGTCGGTTCCTCCAAGGTC
PaguaR	GACGTTGTGGTGCGACTTGA	GTCTCGTGGGCTCGGAGATGTGTATAAGAGACAGCGACGTTGTGGTGCGACTTGA
PamutF	AGAAGACCGAGTTCGACCAT	TCGTCGGCAGCGTCAGATGTGTATAAGAGACAGCCGAGAAGACCGAGTTCGACCAT
PamutR	GGTGCCATAGAGGAAGTCAT	GTCTCGTGGGCTCGGAGATGTGTATAAGAGACAGGCAGGGTGCCATAGAGGAAGTCAT
PanuoF	ACGGCGAGAACGAGGACTAC	TCGTCGGCAGCGTCAGATGTGTATAAGAGACAGCCACGGCGAGAACGAGGACTAC
PanuoR	TGGCGGTCGGTGAAGGTGAA	GTCTCGTGGGCTCGGAGATGTGTATAAGAGACAGTGGCGGTCGGTGAAGGTGAA
PappsF	GGTGACGACGGCAAGCTGTA	TCGTCGGCAGCGTCAGATGTGTATAAGAGACAGGGTGACGACGGCAAGCTGTAC
PappsR	GTATCGCCTTCGGCACAGGA	GTCTCGTGGGCTCGGAGATGTGTATAAGAGACAGGGTATCGCCTTCGGCACAGGA
PatrpF	TTCAACTTCGGCGACTTCCA	TCGTCGGCAGCGTCAGATGTGTATAAGAGACAGCTTTTCAACTTCGGCGACTTCCATGT
PatrpR	GGTGTCCATGTTGCCGTTCC	GTCTCGTGGGCTCGGAGATGTGTATAAGAGACAGCGGTGTCCATGTTGCCGTTCC

Table S7. PCR conditions

Loci	PCR reagent	Ta
S. aureus		
arc	Q5 (NEB)	61
aro	Q5 (NEB)	56
glp	Q5 (NEB)	56
gmk	Phusion (NEB)	56
pta	Q5 (NEB)	61
tpi	Q5 (NEB)	61
yqi	Q5 (NEB)	61
P. aeruginosa		
arc	Kapa (Roche)	59
gua	Kapa (Roche)	59
mut	Q5 + GC buffer (NEB)	55
nuo	Kapa (Roche)	62
pps	Q5 + GC buffer (NEB)	67
trp	Q5 + GC buffer (NEB)	67
